# Catatonia-related adverse outcomes after long-acting injectable antipsychotics: Case series

**DOI:** 10.1177/2050313X241229008

**Published:** 2024-01-31

**Authors:** Joseph D Dragonetti, Jacqueline G Posada, Richard Garrett Key, Joseph L Kugler

**Affiliations:** Department of Psychiatry & Behavioral Sciences, The University of Texas at Austin Dell Medical School, Austin, TX, USA

**Keywords:** Catatonia, schizophrenia, antipsychotic, adverse drug reaction

## Abstract

Due to a lack of controlled, prospective trials examining the pathophysiology and treatment of catatonia, current guidelines vary regarding how and when to best use antipsychotics in the presence of catatonia and what factors to consider in a thorough risk–benefit analysis. The literature is especially limited in describing the risks and benefits of using long-acting injectable antipsychotics in the presence of catatonia. We describe four cases where patients with preexisting catatonia received long-acting injectable first generation antipsychotics and experienced severe adverse effects (three experienced worsening of catatonia and one experienced neuroleptic malignant syndrome). The evidence base for managing comorbid catatonia and psychosis remains underdeveloped and inconsistent, but there are numerous known risk factors for adverse antipsychotic reactions, which we describe in relation to these cases. Finally, we present best practices to consider when managing comorbid psychosis and catatonia, especially when considering the use of long-acting injectable antipsychotics.

## Introduction

Catatonia is a neuropsychiatric syndrome with an estimated prevalence of 10% for psychiatric inpatients and up to 5.5% in medical/surgical inpatients seen by psychiatric consultants.^1,2^ Highly morbid and potentially life-threatening, the syndrome is very important to recognize and treat. The most common associated diagnoses are mood disorders, psychotic disorders, and neurologic disorders.^1,2^ Drug-induced catatonia is another important but underrecognized diagnosis to consider.^3^ Although the pathophysiology and treatment of catatonia remain incompletely understood, benzodiazepines and electroconvulsive therapy (ECT) are well-established first line treatments.^4^ Numerous other agents have been proposed as alternatives, including glutamatergic agents, although substantiating data remains limited.^4^ Antipsychotic medications are common treatments for many underlying conditions associated with catatonia, including psychotic disorders, bipolar disorders, major depressive disorders, and delirium. However, due to a lack of controlled, prospective trials examining the pathophysiology and treatment of catatonia, current guidelines vary regarding how and when to best use antipsychotics in the presence of catatonia and what factors to consider in a thorough risk–benefit analysis. The literature is especially limited in describing the risks and benefits of using long-acting injectable (LAI) antipsychotics in the presence of catatonia.

The neurobiology of catatonia is still not fully understood, but it is known to be complex. Incomplete pathophysiologic understanding of catatonia further complicates its treatment. Catatonia can be conceptualized as dysfunction in motor circuits leading to psychomotor abnormalities, which are often categorized as hypoactivity or hyperactivity.^5^ Accordingly, the thalamus and basal ganglia are believed to have an important role in the pathophysiology of catatonia.^5^ Neuroimaging studies have identified other important areas involved in its pathophysiology including the primary motor cortex (M1), the supplementary motor area, and the pre-supplementary motor area.^5^ Their interactions with the thalamus and basal ganglia within motor circuits serve to mediate motor control, movement initiation, inhibition, and timing.^5^ Functional neuroimaging studies support the involvement of these motor areas via excessive inhibition of the thalamus by the globus pallidus, mediated by the inhibitory neurotransmitter gamma-aminobutyric acid (GABA).^6^ The effective treatment of catatonia with benzodiazepines, and potential detrimental effect of dopamine blockade from antipsychotic medications is likely related to the relationship between dopamine neurons and GABA-A receptors in the basal ganglia, which can decrease dopamine release in the motor circuit.^6^ Further functional neuroimaging studies have also identified involvement of the frontoparietal network including the orbitofrontal cortex, medial prefrontal cortex, and dorsolateral prefrontal cortex, which could help explain the emotional disturbances of catatonia such as impulsivity and agitation.^7,8^ As no single brain region or circuit is fully responsible for the pathophysiology of catatonia, it can be understood as dysfunctions across numerous brain circuits, most specifically the cortico-striatal-thalamic loop which is mediated by dopamine, and the frontoparietal and sensorimotor networks which are mediated by GABAergic and glutamatergic neurons.^8,9^ Understanding the involved neurocircuits can help guide treatment, especially when benzodiazepines are not effective or ECT is not available.

We present a series of cases in which patients with catatonia were administered LAI antipsychotics and subsequently developed worsening catatonia or neuroleptic malignant syndrome (NMS). We describe their initial presentations, relevant comorbidity, and treatment courses. In the discussion, we review risk factors for adverse antipsychotic reactions as well as individual pathophysiologic and systemic contributions to each case. Finally, we summarize the review of relevant literature and suggest best practices to mitigate the risk of catatonia-related adverse outcomes when using LAI antipsychotics.

## Case presentations

Mrs. A is a 55-year-old female with a history of schizophrenia, catatonia, and traumatic brain injury (TBI) who was transferred from a state psychiatric hospital to the general hospital after 6 weeks of poor oral intake and 15 pound weight loss. She received an intramuscular (IM) injection of haloperidol decanoate approximately 8 weeks prior to admission to the general hospital. At that time, she was also prescribed oral (PO) lorazepam 1 mg four times daily (QID) for catatonia, but was not taking it consistently. Upon admission, her initial Bush-Francis Catatonia Rating Scale (BFCRS) score was 14 with immobility/stupor, mutism, negativism, withdrawal, and mitgehen.^10^ Treatment was started with intravenous (IV) fluids and IV lorazepam 1 mg three times daily (TID). Despite 1 week of treatment with IV lorazepam up to 2 mg TID, catatonia persisted, and labs showed signs of dehydration and malnutrition. A nasogastric tube (NGT) was placed for feedings and emergency bitemporal ECT was initiated on hospital day (HD) 10 and continued three times per week. After two sessions of ECT the patient was more interactive and by HD 20 did not require tube feedings. On HD 37, the patient was able to return to the state psychiatric hospital where she continued to receive ECT. After several continuation ECT treatments, her benefit appeared to have plateaued, so ECT was discontinued.

Mrs. B is a 66-year-old female with a history of schizoaffective disorder, bipolar type, catatonia, type 2 diabetes, hypertension, and encephalitis in childhood who presented to the emergency department (ED) with erratic behavior, incontinence, and worsened hallucinations. During preceding months, she had multiple psychiatric hospitalizations for psychosis and catatonia which had responded to PO lorazepam 1 mg TID. One week prior to hospital admission she ran out of lorazepam, and the day prior to admission she received an injection of IM haloperidol decanoate 100 mg at her outpatient clinic. Initial BFCRS in the hospital score was 31 with prominent excitement, impulsivity, combativeness, and dysautonomia. She intermittently required restraints due to impulsivity and combativeness. IV lorazepam was titrated up to 2 mg QID. BFCRS score decreased to eight by HD 10. Sixteen days after discharge she again received IM haloperidol decanoate 100 mg and within 4 days she presented to the ED with confusion, hallucinations, psychomotor agitation, and abnormal posturing. She admitted to not taking her prescribed lorazepam after discharge. She was diagnosed with catatonia and delirium due to hyponatremia. Lorazepam was restarted and valproic acid was added with resulting improvement in catatonia. By HD 8, with her sodium level corrected, her agitation improved, and her thinking was more organized. She left against medical advice before she could be transferred to an inpatient psychiatric hospital. One week later, she was admitted to an inpatient psychiatric facility and her medications were changed to LAI paliperidone and PO diazepam 10 mg at bedtime (QHS) with a resulting increase in stability after discharge.

Mr. C is a 23-year-old male with a history of attention-deficit/hyperactivity disorder and unspecified psychotic disorder who was brought to the ED for 1 week of worsening lethargy, tremor, sialorrhea, and subjective weakness and difficulty moving. He had recently been discharged from an inpatient psychiatric unit after treatment for psychosis, catatonia, and agitation that included two doses of IM fluphenazine decanoate 50 mg, carbamazepine, guanfacine, and benztropine. Oral lorazepam 0.5 mg TID had been trialed but stopped after 3 days. In the ED he developed fever, tachycardia, tachypnea, and lead pipe rigidity. His BFCRS score was 11 with stupor, mutism, staring, withdrawal, and dysautonomia. He was diagnosed with NMS and admitted to the intensive care unit. An elevated creatine kinase level supported the clinical diagnosis of NMS. Treatment was initiated with IV fluids, IV lorazepam, and IV dantrolene. Lorazepam was titrated up to 12 mg per day. Dexmedetomidine and clonidine were used to control agitation and dysautonomia. Due to persistent motor symptoms, he was given a trial of right unilateral ECT three times per week with good initial response allowing for NGT removal. However, improvements plateaued as evidenced by BFCRS score of 13 with stupor, mutism, staring, posturing, and rigidity. Bitemporal ECT was initiated three times per week with a total of 14 ECT treatments administered. Oral lorazepam was tapered to 0.5 mg TID. Physical, occupational, and speech therapy rehabilitated motor symptoms and functional deficits through HD 38, at which time he was stable for discharge home. Two months after hospital discharge, Mr. C’s strength and function had significantly improved, but he continued to have bradykinesia and tremor. His psychiatrist recommended a trial of aripiprazole for treatment of schizophrenia, but the patient and family declined, citing continued hesitance about using antipsychotic medications.

Mr. D is a 59-year-old male with a history of schizophrenia, type 2 diabetes, and recurrent urinary tract infections admitted to the hospital from a correctional facility with several weeks of confusion and decreased responsiveness. He was incarcerated during the 3 months prior to hospital admission, during which time he was started on PO fluphenazine 10 mg daily, and after 1 month required benztropine 1 mg twice daily (BID) for parkinsonism. One week prior to admission he was given an injection of fluphenazine decanoate. In the hospital he was started on oral lorazepam 1 mg TID with oral valproic acid and memantine added as adjunct treatments for catatonia. Lorazepam was increased to 3 mg QID on HD 23 and his BFCRS score decreased from 18 to 9 over the course of 5 days. ECT was recommended for residual catatonia with concern for his nutritional status but the patient lacked capacity to consent. His oral intake was assessed to be sufficient to avoid dehydration and malnutrition so he did not meet criteria for emergent ECT. Lorazepam was tapered beginning on HD 35, reaching a dose of 0.5 mg TID by HD 60, at which time he was discharged to a skilled nursing facility for rehabilitation of strength and mobility.

## Discussion

**Figure 1.**
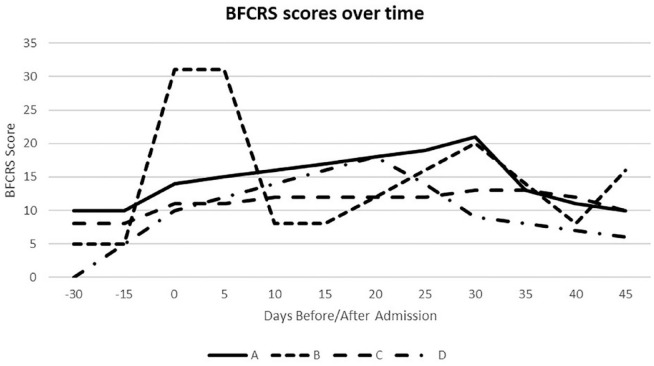
Bush-Francis Catatonia Rating Scale (BFCRS)^37^ scores over time for the four patients presented (some scores estimated based on available history and physical exam if full BFCRS exam was not specifically documented).

This case series describes patients with catatonia who received a LAI antipsychotic for a psychotic disorder and then experienced worsening of catatonia leading to need for weeks to months of inpatient medical care. While the patients’ timelines, symptoms, severity, and treatment responses varied, certain patterns emerge. See Figure 1 for a comparative timeline of the BFCRS scores. In the cases of Mrs. A and Mrs. B, both of whom had prior episodes of catatonia, it is possible that continuing LAI haloperidol in the absence of concurrent benzodiazepines precipitated or accelerated recurrence of catatonia. Although both patients had been diagnosed with catatonia and treated with lorazepam, they were ultimately restarted on haloperidol likely because their primary diagnoses were psychotic disorders. Both also had significant medical histories that may have contributed to their risk for catatonia (Mrs. A had a previous TBI and Mrs. B had a previous episode of encephalitis), highlighting the importance of gathering a full medical history to inform clinical decision making.

In the cases of Mr. C and Mr. D, they each showed poor tolerance of oral fluphenazine, developing parkinsonism that was then treated with benztropine. It has been suggested that drug-induced parkinsonism is the first stage of a spectrum that can progress to drug-induced catatonia and/or NMS, therefore parkinsonism may be an important sign indicating a need to evaluate for catatonia.^11,12^ In retrospect, these side effects overlapped with signs of catatonia that were not fully recognized or treated, but may have benefited from definitive treatment prior to initiation of the LAI antipsychotic, highlighting the importance of maintaining a high index of suspicion for catatonia and ensuring adequate treatment to resolve the syndrome.

The challenges of navigating multiple domains of the healthcare system was another problem each of the four patients encountered. This appeared to impact outpatient follow-up, sharing of medical records, and care coordination among providers and systems. Additionally, all four patients were of lower socioeconomic status, which may have affected their access to certain treatments and their ability to optimally coordinate care. We speculate that the relative cost of different LAI antipsychotics may have led the patients to be treated with first generation antipsychotics (FGA) instead of second generation antipsychotics (SGA). With better care coordination and equitable access to treatments, it is possible that these patients may have experienced fewer or less severe adverse outcomes or that the outcomes could have been identified earlier in the course at a lower level of care.

As a small retrospective study this case series has numerous limitations. All four cases were encountered by consultation-liaison psychiatrists after the patients were admitted to the general hospital. Accordingly, they likely represent the most severe cases by virtue of their need for medical hospitalization. In all four cases, the accuracy of the reported history may have been compromised due to the nature of catatonia and psychosis and collateral history had to be collected from medical records, discussion with other clinicians, and patients’ family members. It was often difficult to pinpoint when specific symptoms began or worsened. In some cases, previous treatments, responses, adherence, and LAI doses could not be verified, and the rationale for selecting specific treatment options (especially first generation LAI antipsychotics) was not known. For most patients, minimal information was available about their progress after discharge from the hospital. Another challenge in systematic evaluation of the cases was inconsistent documentation of complete physical exams or BFCRS scores during the courses of inpatient care. Therefore, the challenges with retrospection and imprecision lead to significant limitations in the conclusions that can be drawn from available data. Finally, the relationship between extrapyramidal symptoms (EPS), catatonia, and NMS continues to be debated, with varying opinions about their degree of overlap and differentiation.^2,13-15^ Here they are presented as separate but related phenomena along a spectrum, which informs the grouping of the cases.

This case series adds to the minimal existing literature concerning the interaction of catatonia with LAI antipsychotics. LAI antipsychotics are increasingly recommended in practice guidelines for treatment of psychotic and bipolar disorders and catatonia is a known comorbidity of these disorders, yet most guidelines do not detail the specific risks and benefits of LAI antipsychotics in the presence of catatonia.^16,17^ Review of five sets of guidelines and three large-scale reviews focused on the use of LAI antipsychotics to treat schizophrenia did not yield any mention of catatonia as an important complication or consideration.^18-25^ Of eight international professional organization guidelines for the treatment of schizophrenia or bipolar disorder, three did not mention catatonia, three briefly mentioned catatonia, and two specifically discussed the interaction between oral antipsychotic medications and catatonia.^16,17,26-31^ Increased awareness and improved understanding of the risks and benefits of using LAI antipsychotics when catatonia is present may allow clinicians to more safely and effectively treat patients.

Existing evidence about the effect of antipsychotics on catatonia is largely based on case reports or series and focuses on oral antipsychotics. FGAs carry a higher risk than SGAs of being associated with antipsychotic-induced catatonia.^14,32^ FGAs also have a known risk of precipitating malignant catatonia or NMS when given in the presence of catatonia.^15^ While there are numerous reported cases of NMS or worsened catatonia caused by SGAs, there are also numerous reports of catatonia being definitively treated with SGAs both with and without benzodiazepines.^1,3,14,33–36^ Overall, it appears reasonable to avoid FGAs as first-line interventions in the presence of catatonia, but the current evidence about risks and benefits of using SGAs in the presence of catatonia is very mixed, especially considering the lack of prospective trials examining this intervention.^14,37^

When reviewing the guidelines and literature for LAI antipsychotics, there is little information specifically describing the risks and benefits of LAI antipsychotics in treating patients with catatonia, largely because there are no controlled trials to reference.^37,38^ Generally, this information has to be inferred based on separate discussions of antipsychotic side effects (such as antipsychotic-induced catatonia or NMS) and the risks and benefits of LAI antipsychotics (wherein tolerance of oral medication and history of severe adverse reactions are important considerations).^17,31^ The relative risk of adverse effects is generally considered equivalent among oral and LAI antipsychotics, with the primary difference in risk relating to the longer elimination time of LAI formulations.^19,24^ Recent reviews of NMS incidence and outcomes have shown that LAI antipsychotics do not infer a higher risk than oral antipsychotics for NMS and in comparing cases of NMS from LAI antipsychotics versus NMS from oral antipsychotics, there were no significant differences in clinical presentation, severity, length of hospital stay, sequelae, or mortality, further supporting risk equivalence of the two antipsychotic formulations.^33,39^ Altogether, there is some literature that peripherally relates to risks and benefits of LAI antipsychotics for patients with catatonia, but this is generally an unexplored and undescribed topic.

Due to the inability to quickly discontinue LAI medications, it is an important standard of care to assess for risk factors for medication intolerance prior to administration. Common risk factors (see Table 1) for adverse reactions to LAI antipsychotics include poor tolerance of the oral formulation, catatonia, EPS, dehydration, and previous brain injuries or brain disease.^11,14,40,41^

**Table 1.** Patient- and antipsychotic-related risk factors for adverse reactions to long-acting injectable antipsychotics.^5,8,34,35^

Patient-related	Antipsychotic-related
Extrapyramidal symptoms Catatonia (current or previous) Low serum iron Young adulthood (age 20–25) Male sex Dehydration Poor tolerance of oral formulation Central dopamine tract lesions Brain disease/injury Prior episode of neuroleptic malignant syndrome	First-generation Strong D_2_ receptor antagonism Polypharmacy Parenteral administration Rapid titration High total dose

Based on the presented cases and a review of the literature about catatonia and antipsychotics, we recommend several practices when utilizing LAI antipsychotics for patients with catatonia (summarized in Table 2). First, maintain an adequate index of suspicion for medication-induced EPS and catatonia and routinely conduct baseline screening in psychiatric inpatients and other high-risk groups using validated and standardized screening tools, such as the Extrapyramidal Symptom Rating Scale (ESRS) and Bush-Francis Catatonia Screening Instrument (BFCSI).^10,42^ When initiating a new antipsychotic, serial assessments can provide valuable information for gauging the patient’s response to the oral formulation, better informing the risk–benefit analysis of using the LAI formulation. Assessing specifically for these syndromes also emphasizes the important distinction between catatonia and psychotic disorders—that they have different manifestations, and, importantly, different treatments.^43^

**Table 2.** Key takeaways for clinical management of comorbid catatonia and psychosis when considering use of a LAI antipsychotic.^37^

1. Routinely screen for EPS and catatonia using standardized instruments (e.g., ESRS, BFCSI) in psychiatric inpatients and patients being started on new antipsychotics. 2. Prioritize definitive, durable treatment of catatonia before transitioning to LAI antipsychotics. 3. For those with elevated risk for catatonia or adverse antipsychotic reactions, avoid first generation LAI antipsychotics without first having tried SGAs. 4. If using a LAI antipsychotic in the presence of catatonia, select a SGA with weaker D_2_ antagonism. 5. Utilize shared decision-making, ensuring that patient and family are thoroughly educated about risks, benefits, and alternatives. 6. Ensure consistent follow-up visits by incorporating family and a multi-disciplinary treatment team.

BFCSI: Bush-Francis Catatonia Screening Instrument; EPS: extrapyramidal symptoms; ESRS: Extrapyramidal Symptom Rating Scale; LAI: long-acting injectable; SGA: second generation antipsychotic.

One of the main takeaways we suggest from this case series is that active catatonia may predispose patients to adverse antipsychotic reactions.^3^ Therefore, erring on the side of caution, it may be worth considering catatonia a relative contraindication to the use of LAI antipsychotics until the syndrome is fully resolved and the patient no longer requires benzodiazepines or ECT for control of catatonia. Oral antipsychotics can be utilized during this time, with the benefit that they can be quickly discontinued if there is concern for worsening catatonia. One study showed very good tolerability of restarting oral antipsychotics after resolution of antipsychotic-induced catatonia.^13^ Nevertheless, it is possible that LAI antipsychotics may appropriately be initiated or continued in the presence of catatonia, but this should be done only in the context of a thorough process of shared decision-making during which the psychiatrist and patient can thoroughly discuss the risks and benefits of, and alternatives to using LAI antipsychotics. This can be a complex discussion when LAI antipsychotics may compensate for poor treatment adherence, having to weigh the risk of experiencing an untreated or undertreated psychotic disorder if not using the LAI formulation against the risk of catatonia-related adverse antipsychotic reactions. If the patient is unable to adequately participate, then family or surrogate decision makers will need to be included in the shared decision-making process.

In cases where the team chooses to use a LAI antipsychotic for a patient with catatonia, best practices to decrease risk for adverse outcomes include concurrent benzodiazepine therapy or ECT, use of a SGA with weaker D_2_ receptor antagonism, and avoiding other pro-catatonic agents.^1,14^ It is best to avoid FGAs with strong D_2_ receptor antagonism (such as haloperidol or fluphenazine) without having first tried SGAs, which carry a lower risk of adverse effects. Finally, coordinated care and consistent follow-up with the treatment team help ensure that adverse effects or complications can be identified early and treatment plans refined accordingly. Patients often require a strong social support system or an engaged multi-disciplinary treatment team for this to happen reliably, further reinforcing the importance of social determinants of health and their interaction with psychopathology.

In order to address the issues highlighted in this series, future research about catatonia must seek to expand upon the limited knowledge about best practices for maintenance treatment of catatonia after the acute syndrome has improved and patients are transitioned to lower levels of care. The role of benzodiazepines and ECT in this time period are of definite interest, but this research must also examine the role of antipsychotics in both acute and maintenance treatment of catatonia. Even small, open label drug trials could help clinicians understand which antipsychotics are more favorable than others for patients with catatonia in terms of both therapeutic and adverse effects. Another area that would benefit from investigation is the relationship among EPS, antipsychotic-induced catatonia, and other forms of catatonia. Improved neurobiological understanding of these processes could help clarify their interrelation (if any) and inform most appropriate interventions. As suggested by this case series, adverse effects from LAI antipsychotics are especially problematic in patients with a history of catatonia, yet their clinical utility is indisputable in populations who struggle with adherence, especially because nonadherence to medications in certain disorders may increase risk for catatonia. There may also be a role for the Food and Drug Administration to release post-marketing surveillance data about LAI antipsychotics and investigate if other patients have experienced catatonia as an adverse effect.

## Conclusion

This literature review highlights the lack of evidence describing clinical factors to consider when using LAI antipsychotics in patients with catatonia. However, we hypothesize that the risk of catatonia associated with LAI antipsychotics may be reduced with the interventions outlined above and in Table 2. This is a topic which could benefit significantly from more studies evaluating pathophysiology, risk factors, and treatment outcomes so that patients and psychiatrists alike can more fully understand the safest and most effective treatments for catatonia and associated disorders.
